# Pediatric Anaphylaxis in a Tertiary Pediatric Emergency Department: Observation Duration and Biphasic Reactions

**DOI:** 10.3390/children13101334

**Published:** 2026-09-30

**Authors:** Murat Ayar, Şule Demir, Cihangir Şahin, Aykut Çağlar

**Affiliations:** 1 Department of Pediatrics, Faculty of Medicine, Aydın Adnan Menderes University, Aydın 09100, Türkiye; 2 Department of Pediatric Emergency Care, Faculty of Medicine, Aydın Adnan Menderes University, Aydın 09100, Türkiye; 3 Department of Pediatric Allergy and Immunology, Faculty of Medicine, Aydın Adnan Menderes University, Aydın 09100, Türkiye

**Keywords:** anaphylaxis, biphasic reaction, pediatric emergency medicine, adrenaline, observation, children

## Abstract

**Highlights:**

**What are the main findings?**

- Adrenaline administration was frequent (94.3%) in this tertiary pediatric emergency department cohort.
- Biphasic reactions were uncommon (4.3%) and all occurred within three hours after initial symptom resolution.

**What are the implications of the main findings?**

- These findings provide real-world pediatric emergency department data on anaphylaxis management and the timing of biphasic reactions.
- Given the small number of biphasic reactions, the findings are descriptive and insufficient to determine the optimal observation duration.

**Abstract:**

**Background/Objectives:** Biphasic reactions are an important consideration during post-treatment observation of children with anaphylaxis; however, real-world pediatric data on observation practices and the timing of these reactions remain limited. This study aimed to describe the clinical characteristics, management practices, observation duration, and occurrence of biphasic reactions among children presenting with anaphylaxis to a tertiary pediatric emergency department (PED). **Methods:** This single-center retrospective observational study included children aged 0–18 years who presented with anaphylaxis to a tertiary PED between January 2014 and December 2024. Potential cases identified through ICD-10 codes and PED intramuscular adrenaline administration records were reviewed to confirm the diagnosis according to established criteria. Demographic and clinical characteristics, suspected triggers, treatments, observation duration, and biphasic reactions were evaluated. **Results:** Seventy anaphylaxis episodes in 68 patients were analyzed. The median age was 93.5 months (IQR, 50–156). Food (32.9%) and insect stings (28.6%) were the most common suspected triggers, followed by drug/vaccine-related triggers (18.6%) and allergen immunotherapy (11.4%). Adrenaline was administered in 94.3% of episodes, and intravenous fluid therapy in 50.0%. Central nervous system involvement and age-adjusted hypotension were more frequent among episodes receiving intravenous fluid therapy (*p* = 0.004 and *p* < 0.001, respectively). The median PED observation duration was 5.5 h (IQR, 2–8). Biphasic reactions occurred in three episodes (4.3%), all within three hours after initial symptom resolution. **Conclusions:** In this tertiary PED cohort, adrenaline administration was frequent, and biphasic reactions were uncommon and occurred during the early observation period. Given the small number of biphasic reactions, these findings should be interpreted as descriptive and are insufficient to determine the optimal observation duration.

## 1. Introduction

Anaphylaxis is a life-threatening medical emergency that can be fatal if not promptly recognized and treated [1]. Although collecting epidemiologic data on anaphylaxis is challenging, reported incidence rates vary widely across countries, partly because of differences in healthcare data sources and surveillance methods, study designs, and case definitions [2]. Nevertheless, many population-based studies have documented an increasing frequency of anaphylaxis over recent decades, attributed to factors such as industrialization, lifestyle changes, and improved recognition by healthcare providers [3,4]. Despite this rise in frequency, mortality rates appear to be declining in countries such as France and Canada, while remaining stable in England, possibly reflecting earlier diagnosis and better adherence to treatment guidelines [5,6,7]. In Türkiye, a recent study by Aytekin et al. [8] found a significant increase in both the frequency and the hospitalization rate, consistent with the literature.

Pediatric anaphylaxis is a common reason for emergency department visits, and pediatric emergency departments (PEDs) play a key role in recognizing, acutely managing, and observing affected children after treatment. Although several international guidelines recommend observation after acute treatment, real-world data on observation practices and the timing of biphasic reactions in pediatric emergency settings remain limited. Most available evidence comes from multicenter registries or population-based datasets, whereas detailed data from individual PEDs are comparatively scarce. Such institutional cohorts may help contextualize guideline recommendations and identify areas where clinical management practices vary.

In addition to prompt initial management, clinicians must remain vigilant for biphasic reactions, defined as the recurrence of anaphylactic symptoms after initial resolution. Although biphasic reactions have been reported up to 72 h after the initial event, available evidence suggests that most occur in the early post-treatment period, and the optimal duration of observation in PEDs remains uncertain. Current international guidelines generally recommend 4–6 h of monitoring for most patients, with longer observation reserved for those with severe presentations, hypotension, or a need for multiple doses of adrenaline [9]. Recent pediatric recommendations further emphasize that observation and discharge decisions should be individualized based on clinical severity, response to treatment, and access to specialist follow-up [10].

Accordingly, the primary objective of this study was to describe the clinical characteristics, management practices, observation duration, and timing of biphasic reactions among children presenting with anaphylaxis to a tertiary PED. Secondary objectives focused on describing treatment patterns, including intravenous fluid administration, as reflections of clinical severity and physician decision-making rather than as indicators of causal or protective effects. This study was neither designed nor powered to identify independent predictors of biphasic reactions or to establish causal relationships.

## 2. Materials and Methods

This was a single-center retrospective observational study conducted in the PED of a tertiary university hospital that served approximately 13,000–28,000 pediatric emergency visits annually during the study period. Pediatric patients aged 0–18 years presenting between 2014 and 2024 were eligible for inclusion.

Potential cases were identified through a broad screening strategy that included ICD-10 codes for anaphylaxis, related allergic reactions, and allergy status (T63.4, T78.0–4, T80.5, T88.6, and Z88.0–9). In addition, PED medication/order records were independently screened for intramuscular adrenaline administration to identify potentially eligible anaphylaxis episodes that may have been assigned an incorrect or nonspecific diagnostic code. All records identified through either screening pathway were subsequently reviewed in detail to determine whether they met the established diagnostic criteria for anaphylaxis as defined by Sampson et al. [1].

**Figure 1:**
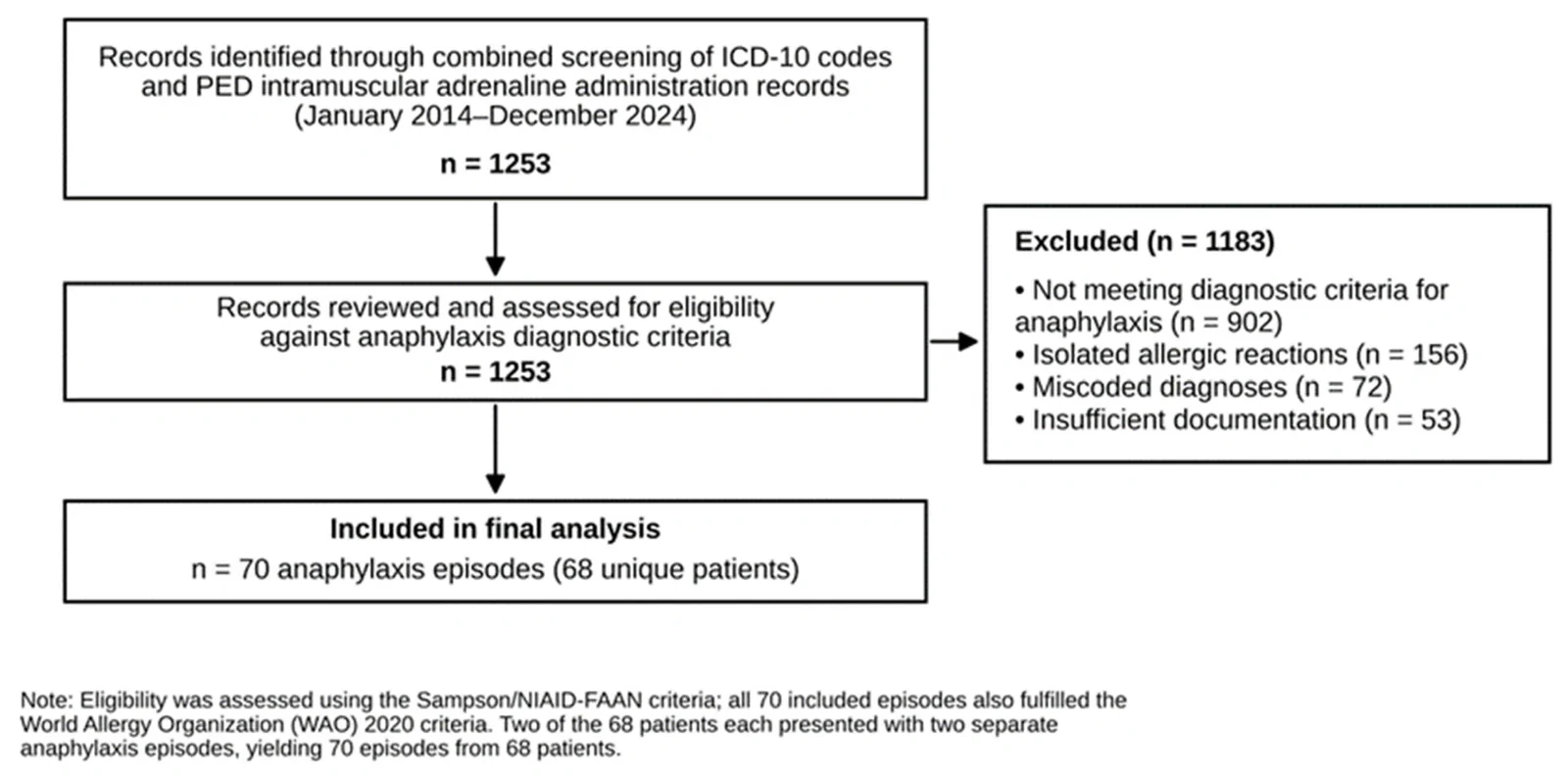
Patient identification and selection process.

A total of 1253 records identified through this combined screening strategy between January 2014 and December 2024 were reviewed. Records were excluded if they did not meet established diagnostic criteria for anaphylaxis, represented isolated allergic reactions without systemic involvement, reflected miscoded diagnoses, or lacked sufficient clinical documentation to confirm anaphylaxis. The numbers of excluded records by major exclusion category are detailed in the patient selection flow chart. After applying the inclusion and exclusion criteria, 70 anaphylaxis episodes from 68 patients were included in the final analysis. No a priori sample size calculation was performed; the study size was determined by the number of eligible anaphylaxis episodes identified during the 11-year study period. The patient identification and selection process is summarized in Figure 1.

Because the registered study protocol specified the World Allergy Organization (WAO) 2020 diagnostic criteria for anaphylaxis [11], eligibility was reassessed using the WAO 2020 definition. In addition to confirming that all 70 included episodes met the WAO 2020 criteria, records previously excluded for not meeting the Sampson criteria were re-evaluated to determine whether any additional episodes met the WAO 2020 criteria. No additional eligible episodes were identified; therefore, the final study cohort remained unchanged at 70 episodes from 68 patients. A biphasic reaction was defined as a recurrence of anaphylactic symptoms after initial clinical resolution, without known re-exposure to the suspected trigger, requiring repeat adrenaline administration within 72 h of the initial episode [9].

The annual frequency of diagnosed anaphylaxis was calculated by dividing the number of anaphylaxis episodes in each calendar year by the total number of PED visits in that year and multiplying by 100,000.

Medical records were reviewed for demographic characteristics, clinical manifestations, suspected triggers, treatments administered in the emergency department, and duration of observation. For trigger classification, vaccines were included in the drug/vaccine-related category, whereas allergen immunotherapy-associated reactions were classified separately. Suspected triggers were identified from the clinical history documented during the index PED visit, based on the temporal relationship between exposure and symptom onset. Subsequent allergy evaluations and confirmatory allergy test results were not systematically collected as part of this retrospective study and were therefore not used for trigger classification. Lengths of PED stay and hospital admission were recorded where applicable. Events occurring outside the index emergency department visit were captured through review of return visits within the institutional medical record system. Post-discharge biphasic reactions were identified through return visits recorded in the institutional electronic medical record system within 72 h. Events managed at other institutions or not resulting in re-presentation could not be captured. Missing data were limited and occurred primarily in vital sign measurements, particularly blood pressure variables. No imputation was performed, and analyses were conducted using available complete-case data. The extent and distribution of missing data are summarized in Supplementary Table S1.

This study was originally designed as a retrospective analytic cohort to evaluate observation practices and potential risk factors for biphasic reactions. However, because only three biphasic reactions were identified during the study period, the planned inferential analyses were not feasible. Therefore, the present report primarily focuses on descriptive and exploratory analyses of anaphylaxis episodes, observation duration, and clinical outcomes.

Statistical analyses were conducted using R (version 4.3.3; R Foundation for Statistical Computing, Vienna, Austria). Data distribution was assessed with the Shapiro–Wilk test. Normally distributed continuous variables were summarized as mean ± standard deviation, whereas non-normally distributed variables were summarized as median and interquartile range (IQR, 25th–75th percentiles). Categorical variables were compared using the chi-square test or Fisher’s exact test, as appropriate. Normally distributed continuous variables were compared using Student’s *t*-test, whereas non-normally distributed continuous variables were compared using the Mann–Whitney U test. *p* values were reported to describe the strength of observed differences and were not used to infer causality, independent prediction, or treatment effects. Given the very small number of biphasic reactions, all statistical comparisons were considered descriptive and exploratory, and no analyses were intended to identify predictors, causal relationships, or protective effects. Multivariable logistic regression analyses were planned in the original study protocol to evaluate potential predictors of biphasic reactions and prolonged observation. However, because only three biphasic reactions were observed and the revised analysis was framed as descriptive and exploratory, these planned risk-factor analyses were not performed, as any resulting estimates would have been statistically unstable and potentially misleading. Therefore, no multivariable model was fitted. For exploratory comparisons by intravenous fluid administration, effect estimates were calculated alongside *p* values. For continuous variables, median differences with bootstrap-derived 95% confidence intervals were reported, and for categorical variables, absolute risk differences with 95% confidence intervals were reported. Effect estimates with 95% confidence intervals for exploratory comparisons by intravenous fluid administration are provided in Supplementary Table S2.

This study was approved by the Non-Interventional Clinical Research Ethics Committee of the Faculty of Medicine at Aydın Adnan Menderes University (Approval No: 2025/266). Because this retrospective study used anonymized medical records, informed consent was waived. The study was retrospectively registered on ClinicalTrials.gov (NCT07231393) and is reported in accordance with the STROBE statement and the RECORD recommendations for studies using routinely collected health data. Completed STROBE and RECORD checklists are provided as Supplementary Materials.

## 3. Results

**Figure 2:**
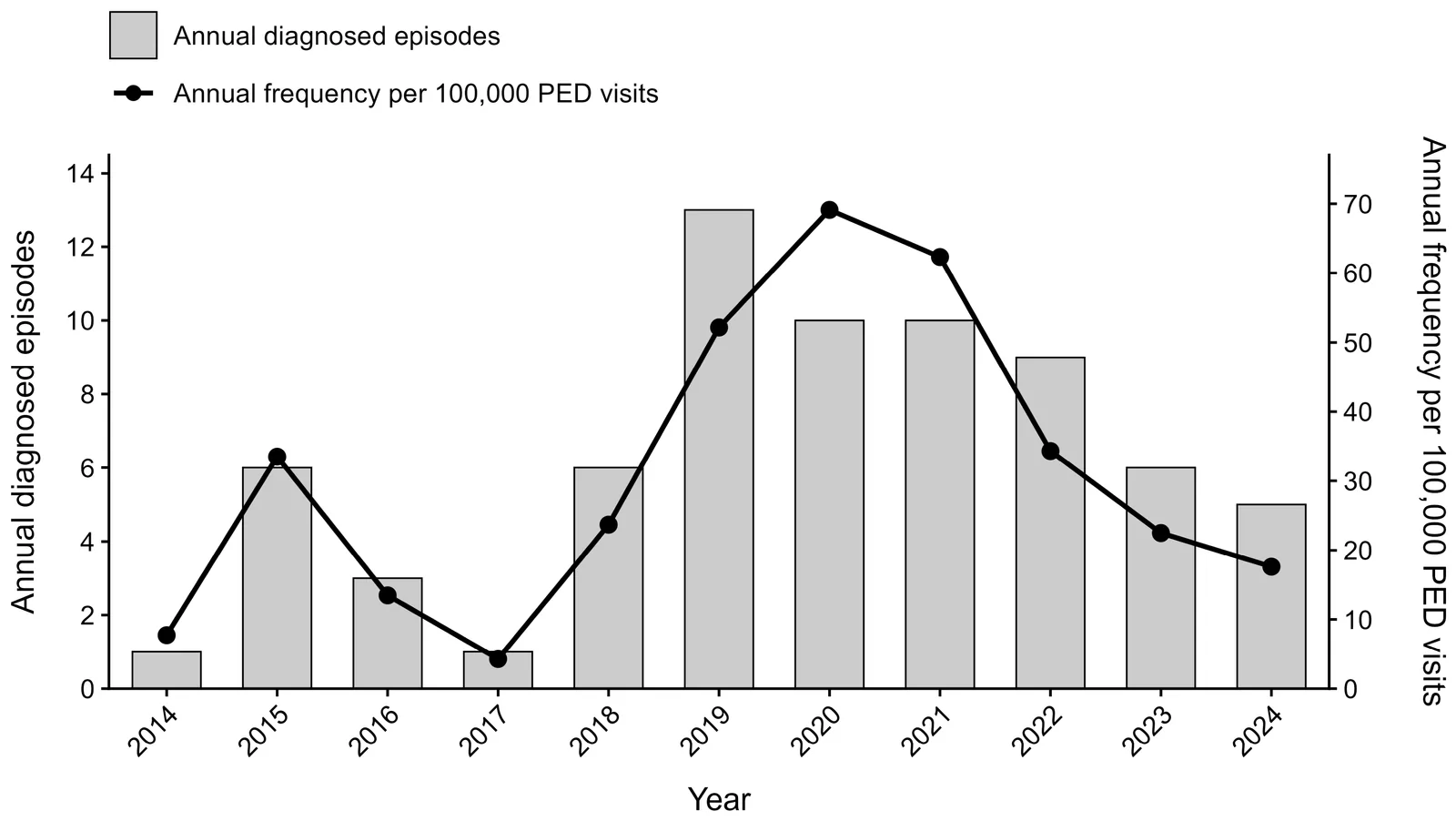
Annual frequency of diagnosed pediatric anaphylaxis in the pediatric emergency department between 2014 and 2024.

A total of 1253 records were reviewed, and 70 anaphylaxis episodes from 68 patients were included in the final analysis (Figure 1). Between 2014 and 2024, the annual frequency of diagnosed anaphylaxis in our PED ranged from 7.7 to 69.1 per 100,000 PED visits, with variation across calendar years (Figure 2).

The male-to-female ratio was 1.19:1. The median age (interquartile range, IQR) was 93.5 (50–156) months, and the median weight across all cases was 30.0 kg (18.0–40.0). Approximately one-third of patients (31.4%) presented during the summer months. The suspected triggers were food in 23 episodes (32.9%), insect stings in 20 (28.6%), drug- or vaccine-related exposures in 13 (18.6%), allergen immunotherapy in 8 (11.4%), and unknown in 6 (8.6%). Among the 23 food-related episodes, the suspected food triggers were cow’s milk (n = 6), egg (n = 6), chicken (n = 2), cocoa (n = 2), walnut (n = 1), wheat (n = 1), green lentil (n = 1), tomato (n = 1), lemonade (n = 1), chocolate (n = 1), and potato chips (n = 1). For composite food products such as lemonade and potato chips, the specific allergenic component could not be determined from the available medical records. Among the 13 drug- or vaccine-related episodes, the suspected triggers were paracetamol (n = 3), ibuprofen (n = 2), ceftriaxone (n = 2), diclofenac sodium (n = 2), metamizole (n = 1), amoxicillin–clavulanate (n = 1), pneumococcal vaccine (n = 1), and cefuroxime (n = 1). Among the eight episodes associated with allergen immunotherapy, the indications for immunotherapy were allergic rhinoconjunctivitis (n = 2), allergic asthma with rhinoconjunctivitis (n = 1), pollen allergy (n = 3), and venom allergy (n = 2).

**Figure 3:**
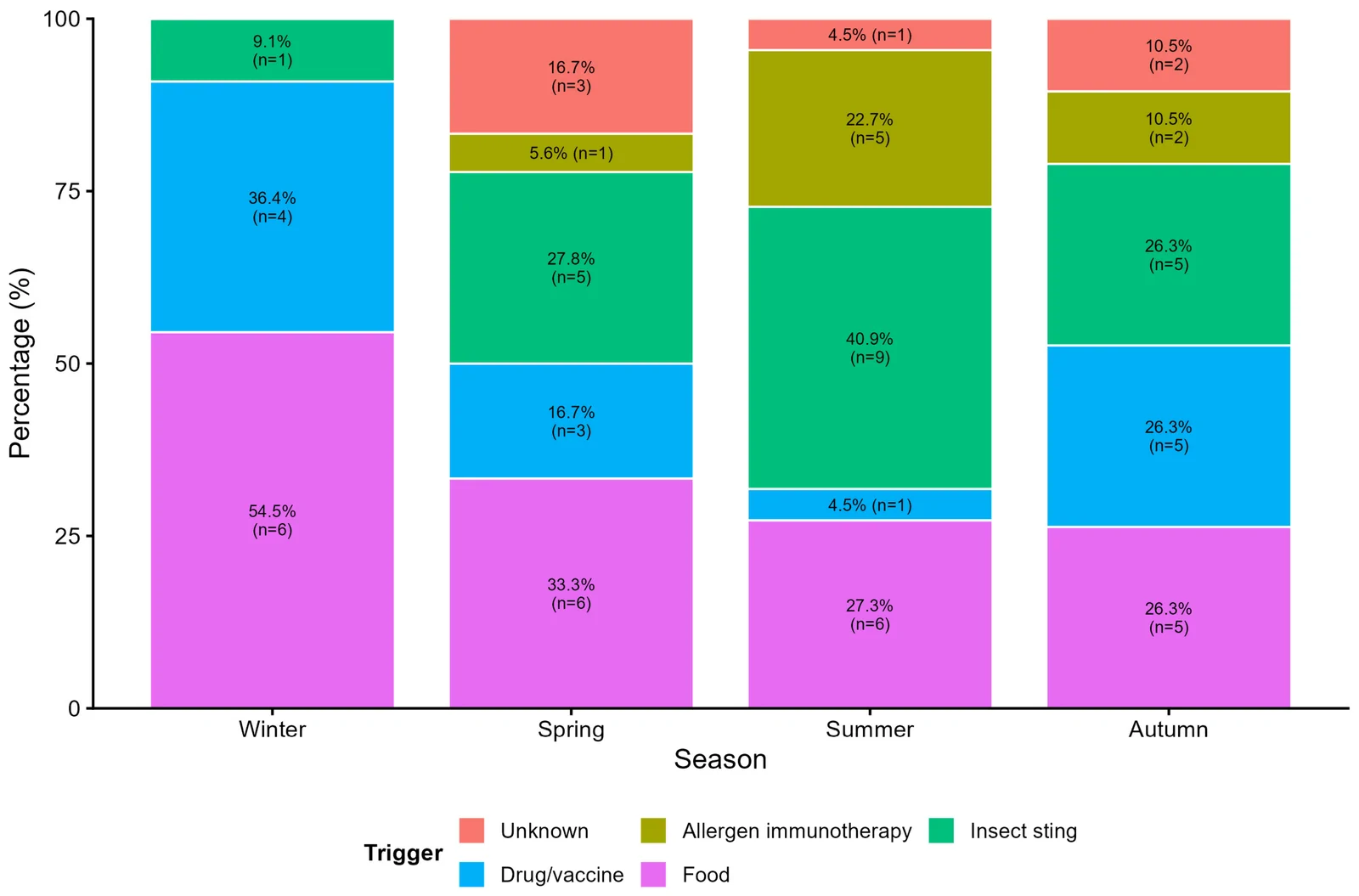
Seasonal distribution of anaphylaxis triggers.

Food was the most frequent trigger in winter presentations (54.5% of winter episodes) and accounted for 33.3% of spring presentations. Food-related episodes were distributed relatively evenly across seasons (n = 6, 6, 6, and 5 in winter, spring, summer, and autumn, respectively). Insect-sting-related anaphylaxis was most prominent during summer, accounting for 40.9% of summer presentations. Allergen immunotherapy-associated episodes occurred predominantly in summer (5/8 episodes), whereas drug/vaccine-related episodes were distributed across spring (n = 3), summer (n = 1), autumn (n = 5), and winter (n = 4) (Figure 3). Detailed demographic and clinical characteristics of the patients are summarized in Table 1.

Most patients had cutaneous (87.1%) and respiratory (57.1%) findings, followed by gastrointestinal (55.7%), cardiovascular (47.1%), and central nervous system (31.4%) manifestations. The median number of involved systems was 3 (2–4), and more than 60% had involvement of three or more systems.

Adrenaline and corticosteroids were the most commonly administered treatments, accounting for 94.3% of episodes, followed by antihistamines (84.3%). Of the 66 episodes treated with adrenaline, 54 (81.8%) received one dose, 11 (16.7%) received two doses, and one (1.5%) received three doses. All adrenaline administrations were intramuscular; no intravenous adrenaline was administered. Intravenous fluid therapy was used in 50.0% of episodes. When clinical characteristics were compared by intravenous fluid administration, episodes receiving intravenous fluid therapy more often had central nervous system involvement and age-adjusted hypotension (*p* = 0.004 and *p* < 0.001, respectively). The absolute risk differences were 34.3 percentage points (95% CI, 14.1 to 54.5) for central nervous system involvement and 33.3 percentage points (95% CI, 16.5 to 50.2) for age-adjusted hypotension. Among episodes receiving intravenous fluid therapy, the median systolic blood pressure was 12 mmHg lower (95% CI, −30 to −2), and the median diastolic blood pressure was 12 mmHg lower (95% CI, −21 to −1). These findings were interpreted as reflecting greater initial clinical severity and clinician-driven treatment decisions rather than any protective or causal effect of intravenous fluid administration. Detailed comparisons of episodes by intravenous fluid therapy status are summarized in Table 2.

In the study group, the median observation duration in the PED was 5.5 h (2–8). A biphasic reaction occurred in 3 of 70 episodes (4.3%; exact 95% CI, 0.9–12.0%). All three patients developed recurrent symptoms within three hours of initial symptom resolution. None received intravenous fluid therapy during initial management. In all cases, adrenaline was administered during the initial episode and repeated during the biphasic reaction. Given the small number of events, these findings are presented descriptively and were not analyzed as predictors of biphasic reactions. Detailed patient characteristics are summarized in Table 3.

## 4. Discussion

The relatively small number of confirmed anaphylaxis episodes should be interpreted in the context of the case-identification strategy. The initial 1253 records represented a deliberately broad screening pool identified through a combination of ICD-10 codes encompassing anaphylaxis, related allergic reactions, and allergy-status codes, together with independent screening of PED intramuscular adrenaline administration records. Therefore, these records should not be interpreted as 1253 patients initially diagnosed with anaphylaxis. After detailed clinical review, most records did not fulfill established diagnostic criteria for anaphylaxis or represented isolated allergic reactions, miscoded diagnoses, or insufficiently documented events. This broad screening approach was intended to maximize identification of potentially eligible cases rather than to estimate the accuracy of anaphylaxis coding. Direct comparison of case frequencies across studies is challenging because published estimates vary according to study population, healthcare setting, case-identification methods, and diagnostic criteria. In our study, all potentially eligible records underwent individual clinical review against predefined anaphylaxis criteria; therefore, the relatively low frequency of confirmed cases may partly reflect methodological differences in case ascertainment rather than a genuinely lower occurrence of anaphylaxis in the underlying population. Importantly, re-evaluation of records excluded under the original Sampson/NIAID-FAAN criteria using the WAO 2020 criteria did not identify any additional eligible episodes, suggesting that the relatively small cohort was not explained solely by overly restrictive diagnostic criteria.

Although the annual frequency of diagnosed anaphylaxis in our PED varied over the study period, these temporal changes should be interpreted cautiously. In this single-center retrospective study, year-to-year variation may reflect changes in referral patterns, healthcare utilization, diagnostic coding, or clinician awareness, in addition to possible changes in the occurrence of anaphylaxis. Therefore, these findings should be considered descriptive and should not be interpreted as population-based incidence trends.

The demographic characteristics of our cohort align with the existing literature indicating a slight male predominance in pediatric anaphylaxis cases [4,7,12]. Seasonal variation in triggers also mirrored patterns reported in major European and Turkish cohort studies [13,14]. Differences in the timing of food-, drug-, and venom-related reactions across the year underscore the clinical relevance of environmental exposures and seasonal factors and highlight the heterogeneity of pediatric anaphylaxis presentations. In addition, the presence of unidentified triggers in a small proportion of our cohort highlights the ongoing diagnostic challenges of pediatric anaphylaxis and reinforces the importance of structured allergy evaluation and follow-up, as emphasized in recent practical recommendations for pediatric idiopathic anaphylaxis [15].

After separating allergen immunotherapy-associated reactions from the drug/vaccine category, drug/vaccine-related exposures accounted for 13 episodes (18.6%), while allergen immunotherapy accounted for 8 episodes (11.4%). Among the drug/vaccine-related episodes, antibiotics and analgesic/anti-inflammatory agents were the most frequently represented drug groups, broadly consistent with a recent multicenter pediatric study from Türkiye in which antibiotics and non-steroidal anti-inflammatory drugs were the predominant triggers of drug-related anaphylaxis [16]. Allergen immunotherapy-associated reactions represented a distinct exposure context in our cohort and were therefore reported separately from drug/vaccine-related anaphylaxis.

Clinical manifestations in our population were consistent with the well-defined pediatric anaphylaxis phenotype, typically involving multisystem involvement. Cutaneous and respiratory symptoms were the most frequent clinical manifestations, underscoring their recognized value in early diagnosis [4,7,17]. Multiorgan involvement highlights the potential severity of anaphylaxis and reinforces the need for rapid, targeted intervention [1,18].

From a treatment perspective, adrenaline was administered in 94.3% of episodes, a higher proportion than reported in many pediatric series [2,8,19]. This high treatment rate may reflect both adherence to guideline-based management and differences in clinical severity or case mix in our tertiary PED. In addition, given the retrospective case-identification strategy, selection bias cannot be excluded, as less overt anaphylaxis presentations may have been under-recognized or missed, potentially contributing to the high observed rate of adrenaline administration. In our cohort, age-adjusted hypotension was present in 16.9% of episodes with available blood pressure measurements, and 18.2% of adrenaline-treated episodes required two or more intramuscular doses; however, no patient required intubation, non-invasive ventilation, intravenous adrenaline, or vasopressor infusion. These findings indicate that clinically significant manifestations were present in a subset of patients but do not establish that the overall cohort was more severe than those of previous studies. This remains consistent with recommendations from the World Allergy Organization and the European Academy of Allergy and Clinical Immunology, which emphasize early intramuscular adrenaline as the cornerstone of anaphylaxis management [2,18]. The frequent use of antihistamines, corticosteroids, and intravenous fluids further reflects common supportive practices, although the relative contribution of these adjunctive treatments to clinical outcomes remains uncertain.

Among children receiving intravenous fluid therapy, central nervous system involvement and age-adjusted hypotension were more common, suggesting that these clinical features may influence decisions about circulatory support. Previous studies have reported substantial variation in intravenous fluid administration in anaphylaxis [8,19,20]. The higher fluid utilization rate observed in our cohort (50%) may reflect differences in institutional practice patterns, patient case-mix, or thresholds for initiating volume resuscitation, rather than evidence of a distinct or more aggressive treatment strategy. Current international guidelines emphasize the importance of intravenous fluid resuscitation in the presence of hypotension or circulatory compromise as part of supportive anaphylaxis management. In this context, our findings highlight potential inter-center variability in the interpretation and management of circulatory findings. Pathophysiologically, capillary leak and intravascular volume depletion are recognized components of severe anaphylaxis and may contribute to hypotension and impaired tissue perfusion; however, the relationship between these mechanisms and subsequent outcomes, including biphasic reactions, remains incompletely defined. Consistent with European and North American reports, clinicians in many settings appear to prioritize prompt adrenaline administration while adopting a more selective approach to intravenous fluid use [19,21]. Because intravenous fluid administration was determined by the treating physician according to the patient’s clinical severity, these exploratory comparisons are subject to confounding by indication and should not be interpreted as evidence of treatment effects. Taken together, these observations emphasize clinical heterogeneity rather than supporting firm conclusions regarding the effect of fluid therapy on anaphylaxis outcomes.

Only three biphasic reactions were observed in our cohort. Therefore, findings related to biphasic reactions should be interpreted cautiously and viewed primarily as descriptive observations. The observed biphasic reaction rate of 4.3% (exact 95% CI, 0.9–12.0%) was broadly consistent with rates reported in previous studies, including the recent study by Rueter et al., which reported biphasic reactions in 4.6% of infants and young toddlers with anaphylaxis [21,22,23]. Similar findings have also been reported in a recent multicenter pediatric study of drug-induced anaphylaxis, in which biphasic reactions remained uncommon despite a large sample size [16]. However, the precision of this estimate remains limited because of the very small number of observed reactions. Because only three biphasic reactions were observed, reliable characterization of their severity or clinical pattern was not possible. All biphasic reactions occurred within the first three hours after initial symptom resolution, a finding that aligns with existing literature [21,24]. Recent large-scale pediatric data have also supported a risk-stratified approach to observation duration, suggesting that shorter monitoring may be reasonable for selected clinically stable children, whereas longer observation remains appropriate for higher-risk presentations [25]. Although this temporal pattern is compatible with current guideline recommendations regarding observation duration, our data are descriptive and insufficient to evaluate the optimal observation duration. Therefore, observation decisions should continue to be individualized according to clinical severity and existing guideline recommendations. Notably, all children who developed biphasic reactions in our cohort had not received intravenous fluid therapy during initial management. Given the very small number of observed biphasic reactions (n = 3), this observation should not be interpreted as implying a protective or causal role of intravenous fluid therapy. Although previous studies have suggested a possible association between inadequate fluid resuscitation and biphasic reactions [26,27], the present data are insufficient to support or refute such hypotheses.

Taken together, these findings highlight the heterogeneity of pediatric anaphylaxis presentations and management practices in routine clinical care. Further prospective multicenter studies are needed to better define optimal supportive management strategies and observation practices. Although our findings largely align with previous pediatric cohorts, they provide real-world data from a tertiary PED and help contextualize observation practices, management patterns, and the timing of biphasic reactions in routine clinical care.

This study has several limitations. The retrospective design is inherently subject to incomplete documentation and potential misclassification, and the single-center setting limits the generalizability of our findings to other healthcare systems or practice environments. In addition, suspected triggers were assigned based on clinical history documented during the index PED visit rather than on systematic confirmatory allergy testing, which may have led to trigger misclassification. Because this was a single-center study, the results may reflect local referral patterns, documentation practices, clinician thresholds for treatment, and institutional observation policies. Therefore, the findings should not be generalized to other healthcare settings without caution. Furthermore, the relatively small overall cohort size remains an important limitation of this study and limits the precision and generalizability of the findings. Although re-evaluation using the WAO 2020 criteria did not identify additional eligible episodes, the sensitivity of the combined case-identification strategy could not be formally assessed, and missed episodes cannot be completely excluded.

Missing data were primarily related to vital signs, particularly blood pressure measurements, which may have affected analyses involving age-adjusted hypotension. Potential under-ascertainment of biphasic reactions is possible because events managed outside our institution or those not resulting in re-presentation could not be captured. Although our institution serves as the main tertiary pediatric referral center for the region, we were unable to determine the proportion of children who may have sought care at other hospitals after discharge; therefore, some biphasic reactions may have remained unrecognized.

Most importantly, the very small number of biphasic reactions substantially limited statistical power. The study was neither designed nor powered to identify predictors of biphasic reactions, and the limited number of events precluded meaningful multivariable modeling or risk factor analyses. No multivariable model was fitted for biphasic reactions because only three events were observed, precluding meaningful adjustment for potential confounders.

## 5. Conclusions

In conclusion, this study provides real-world data on pediatric anaphylaxis management, observation duration, and biphasic reaction timing from a tertiary PED between 2014 and 2024. Our findings are compatible with current guideline recommendations on observation duration; however, given the relatively small cohort and the occurrence of only three biphasic reactions, our data are insufficient to determine the optimal observation duration. Nevertheless, these findings offer real-world PED data that may help contextualize current observation practices and inform the design of future multicenter prospective studies.

## Figures and Tables

**Table 1:** Demographic and clinical characteristics of pediatric anaphylaxis episodes with available data.

Parameter	Available n	Value
Age, months	70	93.5 (50–156)
Sex, female/male, n (%)	70	32 (45.7%)/38 (54.3%)
Weight, kg	69	30.0 (18.0–40.0)
Known allergen history, n (%)	70	29 (41.4%)
Food allergy history, n (%)	70	17 (24.3%)
Drug allergy history, n (%)	70	4 (5.7%)
Insect sting allergy history, n (%)	70	8 (11.4%)
Season of presentation, n (%)	70	
Winter	70	11 (15.7%)
Spring	70	18 (25.7%)
Summer	70	22 (31.4%)
Autumn	70	19 (27.1%)
Trigger type, n (%)	70	
Food	70	23 (32.9%)
Drug/vaccine	70	13 (18.6%)
Insect sting	70	20 (28.6%)
Allergen immunotherapy	70	8 (11.4%)
Unknown	70	6 (8.6%)
System involvement, n (%)	70	
Cutaneous	70	61 (87.1%)
Respiratory	70	40 (57.1%)
Gastrointestinal	70	39 (55.7%)
Cardiovascular	70	33 (47.1%)
Central nervous system	70	22 (31.4%)
Number of involved systems	70	3 (2–4)
Age-adjusted hypotension, n/N (%)	59	10/59 (16.9%)
Treatment administered, n (%)	70	
Adrenaline	70	66 (94.3%)
Antihistamine	70	59 (84.3%)
Corticosteroid	70	66 (94.3%)
Intravenous fluid therapy	70	35 (50.0%)
Oxygen therapy	70	14 (20.0%)
Intubation or non-invasive ventilation	70	0 (0%)
PED length of stay, h	70	5.5 (2–8)
Length of hospital stay, hours	70	24 (12–48)

Data are presented as median (interquartile range) or number/available number (%). Percentages were calculated using available data for each variable. Hypotension was defined using age-adjusted systolic blood pressure thresholds. Abbreviations: IQR, interquartile range; PED, pediatric emergency department.

**Table 2:** Comparison of clinical features and outcomes according to intravenous fluid therapy.

Variable	Total (n = 70)	Fluids (+) (n = 35)	Fluids (–) (n = 35)	*p*
Age, months	93.5 (50–156)	86 (55–162)	96 (46–151)	0.986
Weight, kg	30.0 (18–40)	30.0 (20–40)	28.0 (17–40)	0.782
Heart rate, bpm	118 ± 31.5	116.6 ± 33.2	117.3 ± 30.4	0.807
Systolic BP, mmHg	105 (97–119)	98 (80–109)	110 (100–120)	0.002
Diastolic BP, mmHg	60 (55–74)	57 (50–60)	69 (60–75)	0.001
Age-adjusted hypotension, n/N (%)	10/59 (16.9%)	10/30 (33.3%)	0/29 (0%)	<0.001
Respiratory rate, /min	22 (18–30)	20 (18–29)	22 (18–30)	0.645
Oxygen saturation, %	98 (97–99)	99 (98–99)	98 (97–99)	0.087
Cutaneous findings, n (%)	61 (87.1%)	29 (82.9%)	32 (91.4%)	0.477
Respiratory findings, n (%)	40 (57.1%)	17 (48.6%)	23 (65.7%)	0.227
Cardiovascular findings, n (%)	33 (47.1%)	20 (57.1%)	13 (37.1%)	0.150
Gastrointestinal findings, n (%)	39 (55.7%)	21 (60.0%)	18 (51.4%)	0.631
Central nervous system findings, n (%)	22 (31.4%)	17 (48.6%)	5 (14.3%)	0.004
Number of involved systems	3 (2–4)	3 (2–4)	2 (1–3)	0.024
Adrenaline administered, n (%)	66 (94.3%)	35 (100%)	31 (88.6%)	0.114
Corticosteroid administered, n (%)	66 (94.3%)	33 (94.3%)	33 (94.3%)	1.000
Antihistamine administered, n (%)	59 (84.3%)	29 (82.9%)	30 (85.7%)	1.000
PED length of stay, h	5.5 (2–8)	4 (2–6)	6 (4–10)	0.006
Hospital length of stay, h	24 (12–48)	24 (24–48)	24 (10–48)	0.029

Values are presented as median (interquartile range), mean ± standard deviation, or number/available number (%). Continuous variables were compared using the Mann–Whitney U test or Student’s *t*-test, as appropriate; categorical variables were compared using the chi-square test or Fisher’s exact test. Abbreviations: BP, blood pressure; PED, pediatric emergency department.

**Table 3:** Detailed characteristics of three patients who developed biphasic reactions.

Characteristic	Case 1	Case 2	Case 3
Sex	Male	Male	Male
Age (months)	8	60	76
Trigger	Food (chicken)	Food (egg)	Insect sting (bee)
Systems involved	Skin, CVS, GI, CNS	Skin, CVS	Skin, Respiratory, GI
IV fluid during initial management	No	No	No
Initial adrenaline administration	Outside Hospital	PED	PED
Repeat adrenaline during biphasic reaction	PED	PED	PED
Corticosteroid	Yes	Yes	Yes
Antihistamine	No	No	Yes
PED LOS (h)	12	6	6
Time to biphasic reaction (h)	2	2	3
Hospital LOS (h)	12	18	18

Initial adrenaline administration indicates the location where adrenaline was first administered during the initial anaphylactic episode. Repeat adrenaline during biphasic reaction indicates the location where adrenaline was administered for recurrent symptoms during the biphasic episode. Abbreviations: CNS, central nervous system; CVS, cardiovascular system; GI, gastrointestinal system; LOS, length of stay; PED, pediatric emergency department.

## Data Availability

The datasets used and/or analyzed in the current study are available from the corresponding author upon reasonable request.

## Supplementary Materials

The following supporting information can be downloaded at: https://www.mdpi.com/article/10.3390/children13101334/s1, Supplementary Table S1: Summary of missing data; Supplementary Table S2: Effect estimates with 95% confidence intervals for exploratory comparisons by intravenous fluid administration; STROBE checklist; RECORD checklist [28].
