# Supplementary material for: Pediatric Anaphylaxis in a Tertiary Pediatric Emergency Department: Observation Duration and Biphasic Reactions
Source: Children (Basel). 2026 Sep 30;13(10):1334. doi: 10.3390/children13101334 (PMC13703907; doi:10.3390/children13101334)
Supplement: Supplementary file 1 [file children-13-01334-s001.zip › children-4580319-supplementary.pdf]

Supplementary Table S1. Summary of missing data

Variable	Available n	Missing n	Missing %
Age	70	0	0.0
Weight	69	1	1.4
Sex	70	0	0.0
Season of presentation	70	0	0.0
Anaphylaxis trigger	70	0	0.0
Cutaneous findings	70	0	0.0
Respiratory findings	70	0	0.0
Cardiovascular findings	70	0	0.0
Gastrointestinal findings	70	0	0.0
Central nervous system findings	70	0	0.0
Number of involved systems	70	0	0.0
Heart rate	60	10	14.3
Systolic blood pressure	59	11	15.7
Diastolic blood pressure	59	11	15.7
Respiratory rate	60	10	14.3
Oxygen saturation	60	10	14.3
Age-adjusted hypotension	59	11	15.7
Adrenaline administration	70	0	0.0
Corticosteroid administration	70	0	0.0
Antihistamine administration	70	0	0.0
Intravenous fluid therapy	70	0	0.0
PED length of stay	70	0	0.0
Hospital length of stay	70	0	0.0
Biphasic reaction	70	0	0.0

Supplementary Table S2. Effect estimates with 95% confidence intervals for exploratory comparisons by intravenous fluid administration

A. Continuous variables

Outcome	Median difference (95% CI)	Available n
Age, months	-10 (-29 to 43)	70
Weight, kg	2 (-8 to 14)	69
Heart rate, bpm	13 (-16 to 33)	60
Systolic blood pressure, mmHg	-12 (-30 to -2)	59
Diastolic blood pressure, mmHg	-12 (-21 to -1)	59
Respiratory rate, /min	-2 (-8 to 4)	60
Oxygen saturation, %	1 (0 to 1)	60
Number of involved systems	1 (0 to 1)	70
PED length of stay, h	-2 (-4 to 0)	70
Hospital length of stay, h	0 (0 to 30)	70

B. Categorical variables

Outcome	Risk difference, percentage points (95% CI)	Available n
Cutaneous findings	-8.6 (-24.1 to 7.0)	70
Respiratory findings	-17.1 (-40.0 to 5.7)	70
Cardiovascular findings	20.0 (-2.9 to 42.9)	70
Gastrointestinal findings	8.6 (-14.6 to 31.8)	70
Central nervous system findings	34.3 (14.1 to 54.5)	70
Age-adjusted hypotension	33.3 (16.5 to 50.2)	59

Median differences are expressed as Fluids (+) minus Fluids (−). Risk differences are expressed as absolute percentage-point differences between Fluids (+) and Fluids (−). Positive values indicate higher values or higher frequencies in the Fluids (+) group. Negative values indicate lower values or lower frequencies in the Fluids (+) group.

CI: confidence interval; PED: pediatric emergency department.
